# Hypertension and Dental Implants: A Systematic Review and Meta-Analysis

**DOI:** 10.3390/jcm13020499

**Published:** 2024-01-16

**Authors:** Liljan Hamadé, Salma El-Disoki, Bruno Ramos Chrcanovic

**Affiliations:** 1 Faculty of Odontology, Malmö University, 214 21 Malmö, Sweden; liljan.hamade@gmail.com (L.H.); salma.eldisoki@gmail.com (S.E.-D.); 2 Department of Oral and Maxillofacial Surgery and Oral Medicine, Faculty of Odontology, Malmö University, 214 21 Malmö, Sweden

**Keywords:** dental implant, failure, hypertension, high blood pressure, systematic review, meta-analysis, meta-regression

## Abstract

Purpose: The aim of the present systematic review was to investigate the influence of hypertension on the dental implant failure rate. Methods: An electronic search was undertaken in four databases, plus a manual search of journals. The I^2^ statistic was used to check heterogeneity and the inverse-variance method was used for the meta-analysis. The estimate of relative effect for dichotomous outcome was expressed as an odds ratio (OR). Results: The review included 24 publications. There were 4874 implants (257 failures) placed in hypertensive patients and 16,192 implants (809 failures) placed in normotensive patients. A pairwise meta-analysis showed that implants in hypertensive patients did not have a higher risk of failure than implants placed in normotensive patients (OR 1.100, *p* = 0.671). The log OR of implant failure between hypertensive and normotensive patients did not significantly change with the follow-up time (*p* = 0.824). Conclusions: This review suggests that implants in hypertensive patients do not present higher odds of failure in comparison to normotensive patients. However, further research on this topic, with the use of more rigorous criteria to diagnose patients as being hypertensive, as well as clearer information about the pharmacological management of the condition in the patients, is recommended.

## 1. Introduction

Hypertension or high blood pressure is the condition in which blood pressure is high due to the high force of the circulating blood being continuously elevated in the walls of arteries [1]. The prevalence of hypertension among adults between 30 and 79 years of age in 2019 was 32% in women and 34% in men [2], making it one of the most common chronic medical conditions worldwide and a major cause of premature death worldwide. Its etiology is associated with genetic and behavioral factors, which include older age, obesity, physical inactivity, a diet with a high content of salt, the excessive consumption of alcohol, smoking, etc. [1]. The condition is usually called a “silent killer”, since most people with the condition do not experience any symptoms, and an estimated 46% of hypertensive adults are unaware that they have the condition [1].

Persistent high blood pressure without proper treatment may lead to angina, heart attacks, heart failure, irregular heartbeat, stroke, kidney damage, damage to the heart, and sudden death [1]. Moreover, there is evidence to suggest that the condition is associated with negative effects on bone health due to its connection with many biochemical and physiological pathways [3,4]. Hypertension may also have a negative effect on microvascular remodeling and angiogenesis [5].

The effects of hypertension on bone metabolism and angiogenesis raised questions regarding the condition, which poses a risk regarding oral rehabilitation with dental implants, since these two processes are important for the process of osseointegration, as well as the long-term maintenance of implants into bone [6,7]. The authors of a recent publication evaluated the possible influence of medications on dental implant osseointegration [8]. This study was, however, an umbrella review, without a focus on hypertension. In addition, reviews of this type cannot be completed without pre-existing reviews, and they do not include information regarding epidemiological associations not examined in the included reviews [9]. Another review, but a systematic one, was also published recently, which evaluated the possible effects of the condition on dental implants [10]. Nevertheless, the electronic search was restricted to two databases, and the authors limited the inclusion criteria to studies that reported the type of antihypertensive medication that was used, resulting in only three studies being included. Including such a small number of studies makes it difficult to draw any robust conclusion on the topic. The aim of the present systematic review was, therefore, to test the null hypothesis of there being no difference in the implant failure rates in hypertensive patients in comparison to normotensive patients against the alternative hypothesis of a difference, with more comprehensive inclusion criteria.

## 2. Materials and Methods

This study followed the PRISMA 2020 Statement guidelines [11]. Its registration number in PROSPERO is as follows: CRD42023487489.

### 2.1. Objective

The purpose of the present study was to test the null hypothesis of no difference in the implant failure rates after the insertion of dental implants in patients with hypertension compared to the insertion in non-hypertense patients, against the alternative hypothesis of a difference, based on a systematic review of the literature. The question was elaborated using the participants, interventions, comparisons, outcomes (PICO) format: In patients being rehabilitated with dental implants, what is the effect of hypertension on the implant failure rates in comparison to normotensive patients?

### 2.2. Search Strategies

An electronic search without time restrictions was first undertaken in August 2022, and the last update occurred in October 2023, in the following databases: PubMed/Medline, Web of Science, Scopus, and Ebsco. Due to the high number of initial entries, the search in Scopus was limited to ‘Article’ within the filter ‘Document type’. The following terms were used in the search strategies:

(“dental implant” OR “oral implant”) AND (hypertension OR high blood pressure)

A manual search of dental-implants-related journals was performed (the list of journals can be found in the Supplementary Material). A reference list of the identified studies and the relevant reviews on the subject were also checked for possible additional studies.

### 2.3. Inclusion and Exclusion Criteria

The eligibility criteria included clinical human studies providing information on implant failure rates in hypertense and in normotensive individuals. Only the cases rehabilitated with cylindrical, screw-type, modern dental implants of titanium (c.p.Ti) or its alloys were considered. Exclusion criteria were case reports, technical reports, animal studies, in vitro studies, and review papers. Studies reporting cases rehabilitated with mini-implants, zygomatic, orthodontic, zirconia, subperiosteal, or hollow implants were also excluded.

### 2.4. Study Selection

The titles and abstracts of all reports identified through the electronic searches were read independently by the three authors. For studies appearing to meet the inclusion criteria, or for which there insufficient data were included in the title and abstract to make a clear decision, the full report was obtained. Disagreements were solved by discussion between the authors.

### 2.5. Quality Assessment

A quality assessment of the studies was executed according to the Quality Assessment Tool of the National Institutes of Health [12]. Studies of “good” quality were judged to have at least 7 points.

### 2.6. Definitions

A normal blood pressure level, or normotension, was defined as systolic and diastolic blood pressure lower than 120- and 80-mm Hg, respectively. High blood pressure, or hypertension, was defined as blood pressure equal or higher than 130/80 mm Hg [13].

An implant was considered a failure if signs and symptoms that led to implant removal, i.e., a lost implant, were present. Implant failure could be occur early (due to the host failing to establish or promote osseointegration at the early stages of healing) or late (due to either the failure of the established osseointegration or failure regarding the function of the dental implants) [14]. A fractured implant was also considered a failure [15].

### 2.7. Data Extraction

The following data were extracted: year of publication, study design, country, study setting, number of patients, patients’ age and sex, implant healing period, failed and placed implants in each group, implant system, jaws receiving implants (maxilla and/or mandible), smoking habit, and follow-up time. Authors were contacted to provide missing data was performed.

### 2.8. Meta-Analysis

Implant failure was the dichotomous outcome measure that was evaluated. The statistical unit for ‘implant failure’ was the implant. Whenever the outcomes of interest were not clearly stated, the data were not used for analysis. Heterogeneity was checked using the I^2^ statistic. The inverse variance method was used for the random-effects (heterogeneity *p* < 0.10) or fixed-effects model (heterogeneity *p* ≥ 0.10) [16]. The estimates of a relative effect for dichotomous outcomes were expressed as an odds ratio (OR).

In order to explore the possible heterogeneity of the effect between studies, a meta-regression was performed to verify how the OR was associated with the time of follow-up.

A funnel plot (plot of effect size versus standard error) was drawn. 

The data were analyzed using the statistical software OpenMeta [Analyst], version 64-bit for Windows 10 [17]. The funnel plot was generated with the OpenMEE software, version 64-bit for Windows 10 [18].

## 3. Results

### 3.1. Literature Search

**Figure 1:**
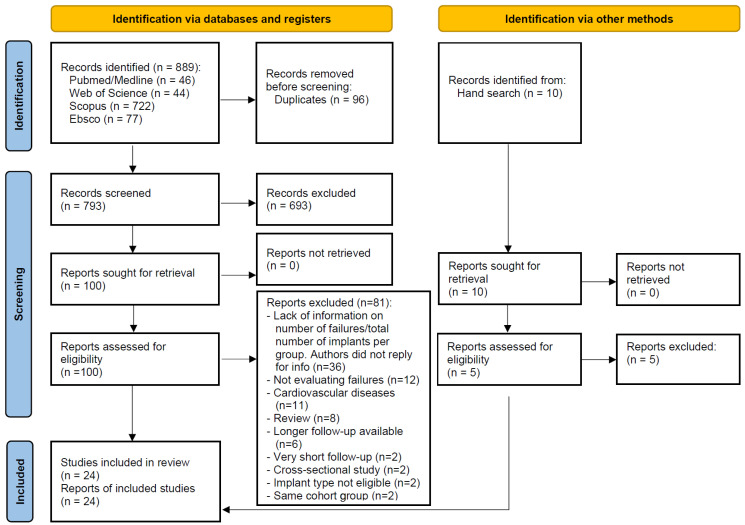
Study screening process.

The search process resulted in 889 papers (46 in PubMed/Medline, 44 in Web of Science, 722 in Scopus, 77 in Ebsco) (Figure 1). After the exclusion of 96 duplicate publications, an additional 693 articles were excluded, as the studies were not related to the subject. Then, 34 studies were excluded due to their containing one or more of the reasons for exclusion. Of the full-text reports of the remaining 66 articles including hypertensive patients (or patients with cardiovascular diseases) that were included in the study, the authors of 51 studies were contacted by e-mail up to three times, asking for missing information that was essential for the analyses, to which the authors of 36 publications did not reply. There was a lack of information concerning either the number of failures or the total number of implants in different groups in these 36 studies, and these publications were therefore excluded from the review. Eleven studies were excluded as they reported patients with “cardiovascular disease”, but did not specify whether or not hypertensive patients were included in this group. Hand-searching yielded 10 articles, of which 5 were eligible. Thus, 24 studies were included in the review. The list of the 81 excluded articles is presented in the Supplementary Material.

### 3.2. Description of the Studies

Table S1 (see Supplementary Material) presents detailed data of the 24 included studies [19,20,21,22,23,24,25,26,27,28,29,30,31,32,33,34,35,36,37,38,39,40,41,42]. The articles were published between 2005 and 2021. Two of the studies were multicenter studies, whereas the other 22 studies were unicenter studies, 8 were prospective, and 16 were retrospective. Sixteen studies were conducted in a university, seven in private practices, and one study in dental public service.

The mean follow-up ± standard deviation of 16 studies was 30.0 ± 21.6 months (min–max, 6–85). For the other eight studies, there was neither information on the precise time of follow-up nor on the mean follow-up time.

Considering the studies, which included patients characterized as presenting hypertension, there were 4874 implants (257 failures) in hypertensive patients and 16,192 implants (809 failures) in normotensive patients.

### 3.3. Quality Assessment

All included studies were classified as “good” according to the quality assessment tool (Table S2, see Supplementary Material). In most cases, the main issues in the publications were related to statistical methods not being well described, and the inclusion of non-consecutive patients in the studies.

### 3.4. Meta-Analysis

**Figure 2:**
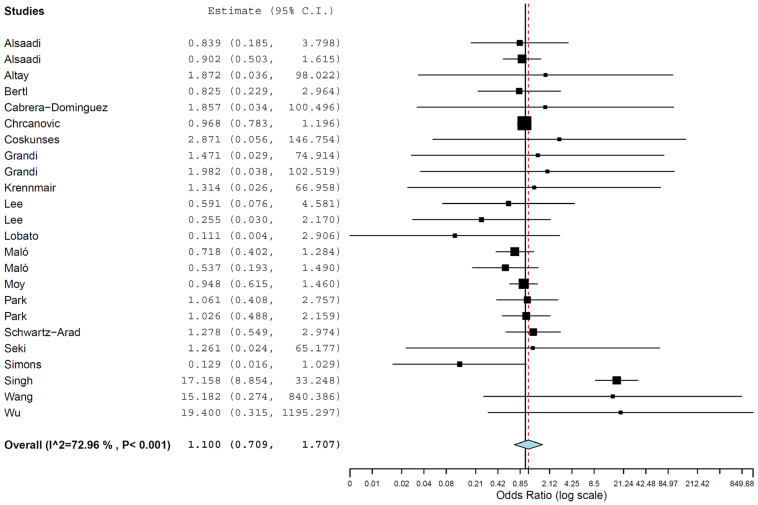
Forest plot for the event ‘implant failure’ [19,20,21,22,23,24,25,26,27,28,29,30,31,32,33,34,35,36,37,38,39,40,41,42].

A random-effects model was used, due to the heterogeneity between the groups of patients (τ^2^ = 0.526, Chi^2^ = 85.063, I^2^ = 72.961, *p* < 0.001). The pairwise meta-analysis showed that implants placed in hypertensive patients did not have a higher risk of failure than implants placed in normotensive patients, with an OR 1.100 (95% confidence interval, 0.709, 1.707, *p* = 0.671; Figure 2).

### 3.5. Meta-Regression

**Figure 3:**
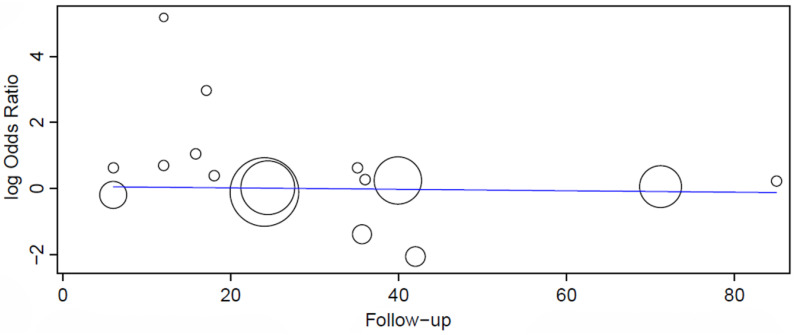
Relationship between the log odds ratio (OR) of implant failure between hypertensive and normotensive patients and the follow-up time (in months). Every circle represents a study, and the size of the circle represents the weight of the study in the analysis. The blue line represents the fitted line plot.

A meta-regression was performed with the 16 studies for which clear information about the follow-up time was provided, with the follow-up included as a covariate in relation to OR. It was observed that the OR did not significantly change with follow-up time (*p* = 0.824) (Figure 3). The linear regression equation of this meta-regression was as follows:

y = 0.065 − 0.002x(1)

where:

Intercept = 0.065 (−0.650, 0.780), standard error 0.365, *p* = 0.858.

Follow-up = −0.002 (−0.021, 0.017), standard error 0.010, *p* = 0.824.

### 3.6. Publication Bias

**Figure 4:**
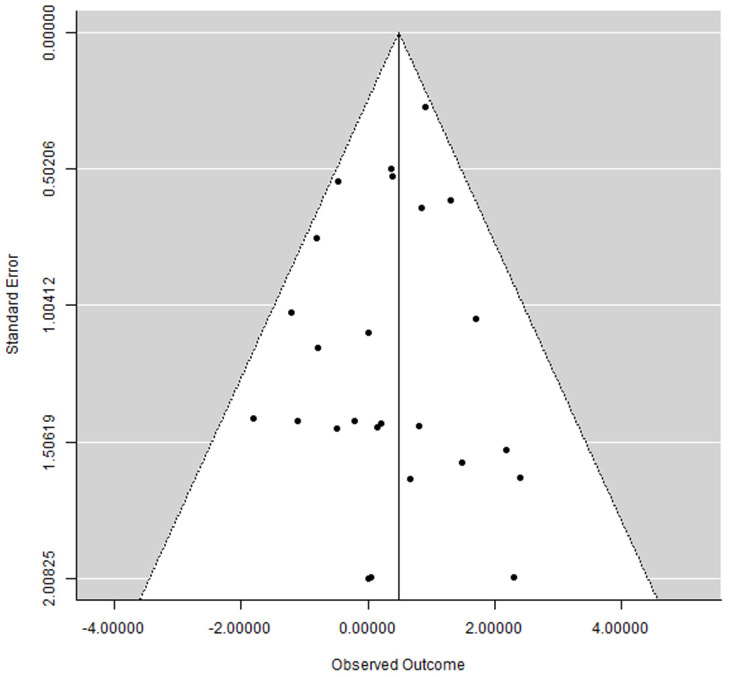
Funnel plot.

The funnel plot did not show clear asymmetry (Figure 4), indicating the possible absence of publication bias.

## 4. Discussion

The purpose of this present systematic study was to compare the failure rate of dental implants within hypertensive to normotensive patients. According to the present results, it is suggested that the use of dental implants in hypertensive patients does not have higher odds of failure than their use in normotensive patients. Therefore, the null hypothesis was not rejected. This may come as a surprise, due to the known effects of high blood pressure on bone metabolism and angiogenesis.

High blood pressure leads to an elevation in the levels of angiotensin II, catecholamines, and the parathyroid hormone (PTH) [43], and the sustained elevation of PTH increases osteoclast formation and differentiation by upregulating the receptor activator of nuclear factor-κβ ligand (RANKL), which plays a critical role in adequate bone metabolism [44], which may contribute to bone resorption. To make matters worse, hypertension is connected to decreased intestinal absorption, increased urinary calcium excretion, and decreased plasma vitamin D concentrations, which all promote the continuous secretion of PTH [43]. The decreased intestinal calcium absorption and increased urinary calcium excretion result in stimulations in the expression of PTH and in increased skeletal calcium mobilization [45]. When it comes to the decreased vitamin D concentration in the plasma, this inhibits the proliferation of vascular smooth cells, vascular calcification, and the renin–angiotensin system [46,47]. Moreover, hypertension may probably result in low bone turnover, one of the mechanisms that could lead to osteoporosis [48]. All these effects have a negative impact on bone mass. More directly related to the dental field, the results of an animal study show that hypertension can alter the expression of receptor activator of nuclear factor-κβ (RANK), RANKL, and osteoprotegerin (OPG, which plays a role in the regulation of bone density), and delay the socket-bone-healing process after tooth extraction., which can exert an influence on some dental procedures, such as implant placement [49]. OPG, RANK, and RANKL are important mediators of bone metabolism, among other cell processes. During the process of bone metabolism, osteoblasts (cells that synthesize bone) modulate osteoclast (a bone cell that breaks down bone tissue) formation and bone resorption by producing OPG and RANKL. OPG then binds to RANKL, preventing it from binding to RANK [50,51], and subsequently inhibiting osteoclast maturation, resulting in the inhibition of osteoclastogenesis as well as lymphocyte development. Alterations in the quality of the mineralized tissue that is formed can occur as a result of any imbalances in the communication between the three factors [52].

Hypertension may also have a negative effect on microvascular circulation and angiogenesis [5]. Hypertension leads to loss of function of capillary endothelium and the constriction of microvessels, which may eventually disappear. High blood pressure has been shown to contribute to the development of microvascular rarefaction in animal models [53,54,55]. The resulting microvascular rarefaction may end up increasing peripheral resistance in the microcirculation, thereby reducing blood flow and reserve, and further elevating blood pressure [56,57,58]. In the microcirculation, the peripheral resistance rises, which, by reducing blood flow, remains normal and continues to increase blood pressure. Major clinical complications due to hypertension include myocardial ischemia, end-organ damage, and stroke, which have been suggested from the results of animal models [5]. Over the short period when hypertension develops, the microvascular refraction worsens or emerges. Hypertension or untreated blood pressure leads to the destruction of capillaries, harming microvessels, and accelerated pathogenic effects are observed in vascular rarefaction [5].

Although a potential factor affecting bone metabolism and microcirculation, and therefore potentially affecting osseointegration and the long-term maintenance of dental implants in the bone, the present results failed to suggest that hypertension may increase the odds of dental implant failure in relation to normotensive patients. This may be a real result, if there actually is no significant effect of hypertension on implant failure rates, or the results may not represent the truth. Unfortunately, there are still unanswered questions due to the lack of rigidity in the studies in classifying patients who have hypertension, if the patients are properly controlled for the condition (namely, the proper intake of anti-hypertensive medication, with effective clinical results), and if the patients presented with other cardiovascular diseases. More information needs to be taken into consideration on patients’ general health status and medication intake in clinical studies on dental implants. It would be unethical, however, to intentionally keep patients in a hypertensive status in order to verify the effect of hypertension on the implant failure rate.

The intake of antihypertensive drugs may be a factor counteracting the negative effects of high blood pressure in relation to bone. The results of some animal studies have suggested that the intake of antihypertensive drugs may reduce bone loss [59,60]. Some clinical studies have shown favorable results when antihypertensive medication is properly and regularly taken by the hypertensive patient, such as it slowing cortical bone loss [61], and having beneficial skeletal effects [62], although the positive benefits on cortical bone density could be small [63]. However, the results of these clinical studies were all observed in either elderly people or postmenopausal women. Other clinical studies failed to observe any benefits [64,65,66], or even observed negative effects of antihypertensive drugs on bone health [67]. Moreover, as previously mentioned, the studies included in the present review did not provide clear information about the controlled status of the patients concerning hypertension, and the majority of them also included young adults. Furthermore, the authors of a recent paper on the subject stated that the current evidence from existing observational studies and randomized trials is not sufficient to establish causal associations for the use of antihypertensive drugs with bone health outcomes [68].

In addition the low specificity concerning hypertension and the intake of antihypertensive drugs, another limitation of the present review includes the presence of confounding factors. For example, many studies reported the presence of a diabetic among the patients, as well as smokers, bruxers, patients submitted to radiotherapy of the head and neck region and/or chemotherapy, and patients taking selective serotonin reuptake inhibitors or proton-pump inhibitors, or bisphosphonates, which are factors that may have a considerable impact on implant failure rates [69,70,71,72,73,74,75,76,77]. Moreover, the implants were placed by different groups of operators, due to the different studies in which they were used, which may also negatively influence implant survival rates, due to variations in the surgeons’ technique, skills, and/or judgment [78]. Furthermore, many studies were retrospective, resulting in incomplete information being obtained, and several studies had small sample sizes and short follow-up periods.

## 5. Conclusions

The findings of the present review suggest that the use of dental implants in hypertensive patients does not present higher odds of failure in comparison to normotensive patients. Further research on this topic, with the use of more rigorous criteria to diagnose patients as being hypertensive, as well as clearer information about the pharmacological management of the condition in patients, is needed.

## Data Availability

All the data resulting from this review are presented in the manuscript.

## Supplementary Materials

The following supporting information can be downloaded at: https://www.mdpi.com/article/10.3390/jcm13020499/s1, List of journals included in the manual (hand) searching; List of excluded articles; Table S1. Detailed data of the included studies; Table S2. Quality assessment of the included studies, according to the National Institutes of Health (NIH).
