# Supplementary material for: Hypertension and Dental Implants: A Systematic Review and Meta-Analysis
Source: J Clin Med. 2024 Jan 16;13(2):499. doi: 10.3390/jcm13020499 (PMC10816909; doi:10.3390/jcm13020499)
Supplement: Supplementary file 1 [file jcm-13-00499-s001.zip › jcm-2809975-supplementary.pdf]

SUPPLEMENTARY MATERIAL

Hypertension and dental implants: a systematic review and meta-analysis

a. **List of journals included in the manual (hand) searching**
b. **List of excluded articles**
c. **Table S1. Detailed data of the included studies**
d. **Table S2. Quality assessment of the included studies, according to the National Institutes of Health (NIH)**

a. List of journals included in the manual (hand) searching

Clinical Implant Dentistry and Related Research, Clinical Oral Implants Research, European Journal of Oral Implantology, Implant Dentistry, International Journal of Implant Dentistry, International Journal of Oral and Maxillofacial Implants, International Journal of Oral Implantology, International Journal of Prosthodontics, Journal of Clinical Periodontology, Journal of Oral Implantology, Journal of Periodontology, Journal of Prosthetic Dentistry, Journal of Prosthodontics, and Journal of Prosthodontic Research

b. List of excluded articles

1. AbdulAzeez AR, Alkinani AA. The Crucial Role of Plaque Control in Peri-Implant Mucositis Initiation as Opposed to the Role of Systemic Health Condition: A Cross-Sectional Study. Clin Cosmet Investig Dent. 2021 Jun 25;13:257-268.
2. Abraha SM, Geng YM, Naujokat H, Terheyden H. Modified Le Fort I interpositional grafting of the severe atrophied maxilla-a retrospective study of 106 patients over 10 years. Clin Oral Implants Res. 2022 Apr;33(4):451-460.
3. Aghaloo T, Pi-Anfruns J, Moshaverinia A, Sim D, Grogan T, Hadaya D. The Effects of Systemic Diseases and Medications on Implant Osseointegration: A Systematic Review. Int J Oral Maxillofac Implants. 2019 Suppl;34:s35-s49.
4. Alsaadi G, Quirynen M, Komárek A, van Steenberghe D. Impact of local and systemic factors on the incidence of oral implant failures, up to abutment connection. J Clin Periodontol. 2007 Jul;34(7):610-7.
5. Alves LB, Coelho TDRC, de Azevedo RA, Dos Santos JN, Neves FS, Cury PR. Systemic risk indicators for peri-implant diseases in individuals with implant-supported fixed prostheses: A cross-sectional study. Int J Oral Implantol (Berl). 2020;13(3):255-266.
6. Anderson N, Lords A, Laux R, Woodall W, Abubakr NH. Retrospective Analysis of the Risk Factors of Peri-implantitis. J Contemp Dent Pract. 2020 Dec 1;21(12):1350-1353.
7. Austin S, Bailey D, Chandu A, Dastaran M, Judge R. Analysis of commonly reported medical conditions amongst patients receiving dental implant therapy in private practice. Aust Dent J. 2015 Sep;60(3):343-52.
8. Bakker MH, Vissink A, Raghoebar GM, Visser A. General health status of Dutch elderly receiving implant-retained overdentures: A 9-year big data cross-sectional study. Clin Implant Dent Relat Res. 2021 Apr;23(2):228-235.
9. Boboeva O, Kwon TG, Kim JW, Lee ST, Choi SY. Comparing factors affecting dental-implant loss between age groups: A retrospective cohort study. Clin Implant Dent Relat Res. 2021 Apr;23(2):208-215.
10. Bornstein MM, Cionca N, Mombelli A. Systemic conditions and treatments as risks for implant therapy. Int J Oral Maxillofac Implants. 2009;24 Suppl:12-27.
11. Bornstein MM, Cionca N, Mombelli A. Systemic conditions and treatments as risks for implant therapy including an update of the literature up to 2015. Implantologie 2015;23(3):313-338.

12. Caban J, Fermergård R, Abtahi J. Long-term evaluation of osteotome sinus floor elevation and simultaneous placement of implants without bone grafts: 10-Year radiographic and clinical follow-up. Clin Implant Dent Relat Res. 2017 Dec;19(6):1023-1033.
13. Cannizzaro G, Felice P, Giorgi A, Lazzarini M, Ferri V, Leone M, Esposito M. Immediate loading of 2 (all-on-2) flapless-placed mandibular implants supporting cross-arch fixed prostheses: interim data from a 1-year follow-up prospective single cohort study. Eur J Oral Implantol. 2012 Spring;5(1):49-58.
14. Carr AB, Arwani N, Lohse CM, Gonzalez RLV, Muller OM, Salinas TJ. Early Implant Failure Associated With Patient Factors, Surgical Manipulations, and Systemic Conditions. J Prosthodont. 2019 Jul;28(6):623-633.
15. Carr AB, Revuru VS, Lohse CM. Association of Systemic Conditions with Dental Implant Failures in 6,384 Patients During a 31-Year Follow-up Period. Int J Oral Maxillofac Implants. 2017 Sep/Oct;32(5):1153-1161.
16. Carr AB, Revuru VS, Lohse CM. Risk of Dental Implant Failure Associated With Medication Use. J Prosthodont. 2019 Aug;28(7):743-749.
17. Chen YW, Simancas-Pallares M, Marincola M, Chuang SK. Grafting and Dental Implantation in Patients With Jawbone Cavitation: Case Series and 3-Year Follow-Up. Implant Dent. 2017 Feb;26(1):158-164.
18. Choi YG, Eckert SE, Kang KL, Shin SW, Kim YK. Epidemiology of Implant Mortality Disparity Among Intraoral Positions and Prosthesis Types. Int J Oral Maxillofac Implants. 2017 May/Jun;32(3):525-532.
19. Chrcanovic BR, Kisch J, Albrektsson T, Wennerberg A. Intake of Proton Pump Inhibitors Is Associated with an Increased Risk of Dental Implant Failure. Int J Oral Maxillofac Implants. 2017 September/October;32(5):1097–1102.
20. Dagorne C, Malet J, Bizouard G, Mora F, Rangé H, Bouchard P. Clinical evaluation of two dental implant macrostructures on peri-implant bone loss: a comparative, retrospective study. Clin Oral Implants Res. 2015 Mar;26(3):307-13.
21. de Souza JG, Neto AR, Filho GS, Dalago HR, de Souza Júnior JM, Bianchini MA. Impact of local and systemic factors on additional peri-implant bone loss. Quintessence Int. 2013 May;44(5):415-24.
22. Dhima M, Paulusova V, Lohse C, Salinas TJ, Carr AB. Practice-based evidence from 29-year outcome analysis of management of the edentulous jaw using osseointegrated dental implants. J Prosthodont. 2014 Apr;23(3):173-81.
23. Di Domênico MB, Farias Collares K, Bergoli CD, dos Santos MBF, Corazza PH, Özcan M. Factors Related to Early Marginal Bone Loss in Dental Implants—A Multicentre Observational Clinical Study. *Applied Sciences*. 2021; 11(23):11197.
24. Diz P, Scully C, Sanz M. Dental implants in the medically compromised patient. J Dent. 2013 Mar;41(3):195-206.
25. Elsubeihi ES, Zarb GA. Implant prosthodontics in medically challenged patients: the University of Toronto experience. J Can Dent Assoc. 2002 Feb;68(2):103-8.
26. Friberg B, Jemt T. Rehabilitation of edentulous mandibles by means of osseointegrated implants: a 5-year follow-up study on one or two-stage surgery, number of implants, implant surfaces, and age at surgery. Clin Implant Dent Relat Res. 2015 Jun;17(3):413-24.
27. Frisch E, Ratka-Krüger P, Wenz HJ. Unsplinted implants and teeth supporting maxillary removable partial dentures retained by telescopic crowns: a retrospective study with >6 years of follow-up. Clin Oral Implants Res. 2015 Sep;26(9):1091-7.
28. Frisch E, Wild V, Ratka-Krüger P, Vach K, Sennhenn-Kirchner S. Long-term results of implants and implant-supported prostheses under systematic supportive implant therapy: A retrospective 25-year study. Clin Implant Dent Relat Res. 2020 Dec;22(6):689-696.
29. Frisch E, Ziebolz D, Ratka-Krüger P, Rinke S. Double crown-retained maxillary overdentures: 5-year follow-up. Clin Implant Dent Relat Res. 2015 Feb;17(1):22-31.
30. Ghaleh Golab K, Balouch A, Mirtorabi S. One-Year Multicenter Prospective Evaluation of Survival Rates and Bone Resorption in One-Piece Implants. Clin Implant Dent Relat Res. 2016 Apr;18(2):392-400.
31. Goiato MC, Santiago Junior JF, Pellizzer EP, Moreno A, Villa LM, Dekon SF, de Carvalho PS, dos Santos DM. Systemic Trans- and Postoperative Evaluations of Patients Undergoing Dental Implant Surgery. Clinics (Sao Paulo). 2016 Mar;71(3):156-62.
32. Göthberg C, André U, Gröndahl K, Ljungquist B, Thomsen P, Slotte C. Immediately loaded implants with or without abutments supporting fixed partial dentures: 1-year results from a prospective, randomized, clinical trial. Clin Implant Dent Relat Res. 2014 Aug;16(4):487-500.
33. Göthberg C, André U, Gröndahl K, Thomsen P, Slotte C. Bone Response and Soft Tissue Changes Around Implants With/Without Abutments Supporting Fixed Partial Dentures: Results From a 3-Year, Prospective, Randomized, Controlled Study. Clin Implant Dent Relat Res. 2016 Apr;18(2):309-22.

34. Hopp M, de Araújo Nobre M, Maló P. Comparison of marginal bone loss and implant success between axial and tilted implants in maxillary All-on-4 treatment concept rehabilitations after 5 years of follow-up. Clin Implant Dent Relat Res. 2017 Oct;19(5):849-859.
35. Hsiao CC, Liang CH, Shen YF, Hsu KW. Retrospective comparison of posterior fixed dental prostheses supported by two different titanium abutments on tissue level implants. J Prosthet Dent. 2021 Jun;125(6):877-882.
36. Khadivi V, Anderson J, Zarb GA. Cardiovascular disease and treatment outcomes with osseointegration surgery. J Prosthet Dent. 1999 May;81(5):533-6.
37. Kim HC, Park SY, Han MS, Lee YM, Ku Y, Rhyu IC, Seol YJ. Occurrence of Progressive Bone Loss Around Anodized Surface Implants and Resorbable Blasting Media Implants: A Retrospective Cohort Study. J Periodontol. 2017 Apr;88(4):329-337.
38. Kim HJ, Yea S, Kim KH, Lee YM, Ku Y, Rhyu IC, Seol YJ. A retrospective study of implants placed following 1-stage or 2-stage maxillary sinus floor augmentation by the lateral window technique performed on residual bone of <4 mm: Results up to 10 years of follow-up. J Periodontol. 2020 Feb;91(2):183-193.
39. Kim JK, Yoon HJ. Clinical and radiographic outcomes of immediate and delayed placement of dental implants in molar and premolar regions. Clin Implant Dent Relat Res. 2017 Aug;19(4):703-709.
40. Kim S, Jung UW, Cho KS, Lee JS. Retrospective radiographic observational study of 1692 Straumann tissue-level dental implants over 10 years: I. Implant survival and loss pattern. Clin Implant Dent Relat Res. 2018 Oct;20(5):860-866.
41. Kim YY, Song YW, Kim MJ, Cha JK, Park JM, Kim JH, Jung UW. Immediate loading of fixed partial prostheses reconstructed using either tapered or straight implants in the posterior area: A randomized clinical trial. Clin Implant Dent Relat Res. 2021 Oct;23(5):703-715.
42. Krennmair S, Weinländer M, Malek M, Forstner T, Krennmair G, Stimmelmayr M. Mandibular Full-Arch Fixed Prostheses Supported on 4 Implants with Either Axial Or Tilted Distal Implants: A 3-Year Prospective Study. Clin Implant Dent Relat Res. 2016 Dec;18(6):1119-1133.
43. Lazarov A. Immediate Functional Loading: Results for the Concept of the Strategic Implant®. Ann Maxillofac Surg. 2019 Jan-Jun;9(1):78-88.
44. Lian M, Zhao K, Wang F, Huang W, Zhang X, Wu Y. Stud vs Bar Attachments for Maxillary Four-Implant-Supported Overdentures: 3- to 9-year Results from a Retrospective Study. Int J Oral Maxillofac Implants. 2019 July/August;34(4):936–946.
45. Lopes A, Maló P, de Araújo Nobre M, Sánchez-Fernández E, Gravito I. The NobelGuide® All-on-4® Treatment Concept for Rehabilitation of Edentulous Jaws: A Retrospective Report on the 7-Years Clinical and 5-Years Radiographic Outcomes. Clin Implant Dent Relat Res. 2017 Apr;19(2):233-244.
46. Lopes A, Maló P, de Araújo Nobre M, Sanchez-Fernández E. The NobelGuide® All-on-4® Treatment Concept for Rehabilitation of Edentulous Jaws: A Prospective Report on Medium- and Long-Term Outcomes. Clin Implant Dent Relat Res. 2015 Oct;17 Suppl 2:e406-16.
47. Malm MO, Jemt T, Stenport VF. Patient factors related to early implant failures in the edentulous jaw: A large retrospective case-control study. Clin Implant Dent Relat Res. 2021 Jun;23(3):466-476.
48. Maló P, de Araújo Nobre M, Lopes A, Ferro A, Gravito I. All-on-4® Treatment Concept for the Rehabilitation of the Completely Edentulous Mandible: A 7-Year Clinical and 5-Year Radiographic Retrospective Case Series with Risk Assessment for Implant Failure and Marginal Bone Level. Clin Implant Dent Relat Res. 2015 Oct;17 Suppl 2:e531-41.
49. Maló P, Nobre Md, Lopes A. Immediate loading of 'All-on-4' maxillary prostheses using trans-sinus tilted implants without sinus bone grafting: a retrospective study reporting the 3-year outcome. Eur J Oral Implantol. 2013 Autumn;6(3):273-83.
50. Mameno T, Wada M, Onodera Y, Fujita D, Sato H, Ikebe K. Longitudinal study on risk indicators for peri-implantitis using survival-time analysis. J Prosthodont Res. 2019 Apr;63(2):216-220.
51. Manor Y, Simon R, Haim D, Garfunkel A, Moses O. Dental implants in medically complex patients-a retrospective study. Clin Oral Investig. 2017 Mar;21(2):701-708.
52. Marchio V, Derchi G, Cinquini C, Miceli M, Gabriele M, Alfonsi F, Barone A. Tissue level implants in healthy versus medically compromised patients: a cohort comparative study. Minerva Stomatol. 2020 Oct;69(5):295-301.
53. Missinne K, Duyck J, Naert I, Quirynen M, Bertrand S, Vandamme K. Oral Implant Restorations By Undergraduate Students: An Up To 5-Years Clinical Outcome. Int J Prosthodont. 2021 July/August;34(4):433–440.
54. Morales-Vadillo R, Leite FP, Guevara-Canales J, Netto HD, Miranda Chaves Md, Cruz F, Cruz G, Cruz-Pierce S, Cruz M. Retrospective study of the survival and associated risk factors of wedge-shaped implants. Int J Oral Maxillofac Implants. 2013 May-Jun;28(3):875-82.

55. Nettemu SK, Nettem S, Singh VP, William SS, Gunasekaran SS, Krisnan M, Abas AL. Multilevel analysis of site, implant, and patient-level factors with peri-implant bleeding on probing: a cross sectional study. Int J Implant Dent. 2021 Jun 10;7(1):77.
56. Neves J, de Araújo Nobre M, Oliveira P, Martins Dos Santos J, Malo P. Risk Factors for Implant Failure and Peri-Implant Pathology in Systemic Compromised Patients. J Prosthodont. 2018 Jun;27(5):409-415.
57. Niedermaier R, Stelzle F, Riemann M, Bolz W, Schuh P, Wachtel H. Implant-Supported Immediately Loaded Fixed Full-Arch Dentures: Evaluation of Implant Survival Rates in a Case Cohort of up to 7 Years. Clin Implant Dent Relat Res. 2017 Feb;19(1):4-19.
58. Nogueira TE, Aguiar FMO, de Barcelos BA, Leles CR. A 2-year prospective study of single-implant mandibular overdentures: Patient-reported outcomes and prosthodontic events. Clin Oral Implants Res. 2018 Jun;29(6):541-550.
59. Okamoto T, Hoshi K, Fukada K, Kataoka T, Kumasaka A, Kaibuchi N, Fukuzawa S, Ando T. Factors Affecting the Occurrence of Complications in the Early Stages After Dental Implant Placement: A Retrospective Cohort Study. Implant Dent. 2018 Apr;27(2):221-225.
60. Parihar AS, Madhuri S, Devanna R, Sharma G, Singh R, Shetty K. Assessment of failure rate of dental implants in medically compromised patients. J Family Med Prim Care. 2020 Feb 28;9(2):883-885.
61. Pedro RE, De Carli JP, Linden MS, Lima IF, Paranhos LR, Costa MD, Bós ÂJ. Influence of Age on Factors associated with Peri-implant Bone Loss after Prosthetic Rehabilitation over Osseointegrated Implants. J Contemp Dent Pract. 2017 Jan 1;18(1):3-10.
62. Romandini M, Cordaro M, Donno S, Cordaro L. Discrepancy between patient satisfaction and biologic complication rate in patients rehabilitated with overdentures and not participating in a structured maintenance program after 7 to 12 years of loading. Int J Oral Maxillofac Implants. 2019 September/October;34(5):1143–1151.
63. Romanos GE, Gaertner K, Nentwig GH. Long-term evaluation of immediately loaded implants in the edentulous mandible using fixed bridges and platform shifting. Clin Implant Dent Relat Res. 2014 Aug;16(4):601-8.
64. Sánchez-Siles M, Ballester-Ferrandis JF, Salazar-Sánchez N, Gómez-García FJ, Moraleja-Ruiz R, Camacho-Alonso F. Long-term evaluation of quality of life and satisfaction between implant bar overdentures and conventional complete dentures: A 23 years retrospective study. Clin Implant Dent Relat Res. 2018 Apr;20(2):208-214.
65. Santhosh Kumar MP. Effects of systemic conditions on dental implants. International Journal of Clinical Dentistry. 2002;15(3):529-538.
66. Saravi B, Vollmer A, Lang G, Adolphs N, Li Z, Giers V, Stoll P. Impact of renin-angiotensin system inhibitors and beta-blockers on dental implant stability. Int J Implant Dent. 2021 Apr 8;7(1):31.
67. Sbricoli L, Bazzi E, Stellini E, Bacci C. Systemic Diseases and Biological Dental Implant Complications: A Narrative Review. Dent J (Basel). 2022 Dec 29;11(1):10.
68. Schimmel M, Srinivasan M, McKenna G, Müller F. Effect of advanced age and/or systemic medical conditions on dental implant survival: A systematic review and meta-analysis. Clin Oral Implants Res. 2018 Oct;29 Suppl 16:311-330.
69. Schlee M, Pradies G, Mehmke WU, Beneytout A, Stamm M, Meda RG, Kamm T, Poiroux F, Weinlich F, del Canto Pingarron M, Crichton E, Poulet JB, Bousquet P. Prospective, Multicenter Evaluation of Trabecular Metal-Enhanced Titanium Dental Implants Placed in Routine Dental Practices: 1-Year Interim Report From the Development Period (2010 to 2011). Clin Implant Dent Relat Res. 2015 Dec;17(6):1141-53.
70. Staedt H, Rossa M, Lehmann KM, Al-Nawas B, Kämmerer PW, Heimes D. Potential risk factors for early and late dental implant failure: a retrospective clinical study on 9080 implants. Int J Implant Dent. 2020 Nov 30;6(1):81.
71. Takamoli J, Pascual A, Martinez-Amargant J, Garcia-Mur B, Nart J, Valles C. Implant failure and associated risk indicators: A retrospective study. Clin Oral Implants Res. 2021 May;32(5):619-628.
72. Testori T, Clauser T, Scaini R, Wang HL, Del Fabbro M. Long-Term Results of Intraforaminal Immediately Loaded Implants and Posterior Mandibular Regrowth Evaluation in Severely Atrophic Mandibles. Int J Oral Maxillofac Implants. 2022 Jan-Feb;37(1):199-207.
73. Testori T, Galli F, Fumagalli L, Capelli M, Zuffetti F, Deflorian M, Parenti A, Del Fabbro M. Assessment of Long-Term Survival of Immediately Loaded Tilted Implants Supporting a Maxillary Full-Arch Fixed Prosthesis. Int J Oral Maxillofac Implants. 2017 Jul/Aug;32(4):904-911.

74. Tonini KR, Hadad H, Egas LS, Sol I, de Carvalho PSP, Ponzoni D. Successful Osseointegrated Implants in Hypertensive Patients: Retrospective Clinical Study. Int J Oral Maxillofac Implants. 2022 May-Jun;37(3):501-507.
75. Trbakovic A, Bongenhielm U, Thor A. A clinical and radiological long-term follow-up study of narrow diameter implants in the aesthetic area. Clin Implant Dent Relat Res. 2018 Aug;20(4):598-605.
76. Ustaoğlu G, Erdal E. Relationship between risk markers for cardiovascular disease and peri-implant diseases. Int J Implant Dent. 2020 Nov 25;6(1):73.
77. van Steenberghe D, Jacobs R, Desnyder M, Maffei G, Quirynen M. The relative impact of local and endogenous patient-related factors on implant failure up to the abutment stage. Clin Oral Implants Res. 2002 Dec;13(6):617-22.
78. Wang F, Monje A, Huang W, Zhang Z, Wang G, Wu Y. Maxillary Four Implant-retained Overdentures via Locator® Attachment: Intermediate-term Results from a Retrospective Study. Clin Implant Dent Relat Res. 2016 Jun;18(3):571-9.
79. Woelber JP, Ratka-Krueger P, Vach K, Frisch E. Decementation Rates and the Peri-Implant Tissue Status of Implant-Supported Fixed Restorations Retained via Zinc Oxide Cement: A Retrospective 10-23-Year Study. Clin Implant Dent Relat Res. 2016 Oct;18(5):917-925.
80. Zhang Y, Chow L, Siu A, Fokas G, Chow TW, Mattheos N. Patient-reported outcome measures (PROMs) and maintenance events in 2-implant-supported mandibular overdenture patients: A 5-year prospective study. Clin Oral Implants Res. 2019 Mar;30(3):261-276.
81. Zuffetti F, Capelli M, Galli F, Del Fabbro M, Testori T. Post-extraction implant placement into infected versus non-infected sites: A multicenter retrospective clinical study. Clin Implant Dent Relat Res. 2017 Oct;19(5):833-840.

c. **Table S1. Detailed data of the included studies.**

Study	Year	Study Design	Country / Setting	Patients (male/female; hypertensive) (n)	Patients' Age Range (mean) (years)	Healing period / loading	Implants location (maxilla and/or mandible)	Were there smokers in the group of patients? [a]	Observations
Alsaadi	2008 a	PS (unicenter)	Belgium / University	283 (96/187; NM)	18-86 (56.2)	Loading was not applied	Mx, Md	Yes, but exact number not informed	Patients with several systemic condition were included, but the exact number not informed
Alsaadi	2008 b	RA (unicenter)	Belgium / University	412 (172/240; 63)	NM	NM	Mx, Md	61 smokers	10 diabetic, 6 rheumatoid arthritis, 2 radiotherapy, 3 chemotherapy, 19 osteoporosis, 25 hypothyroidism, 29 depression
Altay	2018	RA (unicenter)	Turkey / University	13 (6/7; 5)	41-70 (55.2)	Delayed (3-6 mo)	Mx, Md	No	5 diabetic
Bertl	2019	RA (unicenter)	Austria / University	444 (192/252; 198)	65-91 (72)	NM	Mx, Md	48 smokers	47 diabetic, 41 osteoporosis, 31 bisphosphonate, 384 patients with history of periodontitis, 48 taking selective serotonin reuptake inhibitors, 66 taking proton-pump inhibitors
Cabrera-Domínguez	2017	PS (unicenter)	Spain / University	29 (12/17; 10)	NM (55.9)	Delayed (2 mo)	Mx, Md	NM	15 diabetic
Chrcanovic	2017	RA (unicenter)	Sweden / Public service	1406 (625/781; 351)	NM	Immediate, delayed	Mx, Md	285 smokers 33 former smokers	54 bruxers, 95 diabetic, 32 radiotherapy, 18 bisphosphonate, 92 depression, 85 asthma, 58 hypothyroidism
Coskunses	2021	PS (unicenter)	Turkey / University	28 (17/11; 10)	23-72 (52)	Immediate	Mx, Md	6 smokers	2 diabetic, 1 thyroid dysfunction
Grandi	2012 a	PS (multicenter)	Italy / Private practice	47 (22/25; 16)	52-78 (62.3)	Immediate	Md	11 light and heavy smokers	2 diabetic
Grandi	2012 b	PS (multicenter)	Italy / Private practice	42 (13/29; 14)	71-89 (76.5)	Immediate	Md	9 light smokers	2 diabetic
Krennmair	2016	PS (unicenter)	Austria / University	44 (21/23; 19)	NM (62.9)	Delayed (3 mo)	Md	9 smokers	4 diabetic, 6 rheumatic disorders

Lee	2018	RA (unicenter)	Taiwan / Private practice	60 (27/33; 6)	23-90 (53)	Immediate, delayed	Mx, Md	7 smokers	12 history of periodontitis, no diabetic
Lee	2019	RA (unicenter)	South Korea / University	156 (70/86; 60)	19-84 (59.9)	Delayed	Mx, Md	NM	18 diabetic, 21 osteoporosis, 3 chemotherapy, 7 thyroid dysfunction
Lobato	2020	PS (unicenter)	Brazil / University	44 (NM; NM)	25-77 (50.8)	Delayed (4-6 mo)	Mx, Md	4 light smokers 3 heavy smokers	Some diabetic patients, but exact number not informed
Maló	2019 a	RA (unicenter)	Portugal / Private practice	1072 (442/630; 148)	20-88 (55.8)	Immediate	Mx	241 smokers	49 diabetic, 29 osteoporosis, 40 bruxers, 3 depression
Maló	2019 b	RA (unicenter)	Portugal / Private practice	471 (185/286; 47)	20-85 (57.7)	Immediate	Md	117 smokers	11 diabetic, 12 osteoporosis, 90 bruxers
Moy	2005	RA (unicenter)	USA / University	1140 (463/677; 202)	12-94 (58)	NM	Mx, Md	173 smokers	48 diabetic, 22 radiotherapy, 10 chemotherapy, 75 pulmonary disease
Park	2017	RA (unicenter)	South Korea / University	346 (217/129; 149)	65-89 (NM)	NM	Mx, Md	NM	Some diabetic patients, but exact number not informed
Park	2020	RA (unicenter)	South Korea / University	178 (87/91; NM)	19-86 (53)	Delayed	Mx	NM	Some diabetic patients, but exact number not informed
Schwartz-Arad	2016	RA (unicenter)	Israel / Private practice	214 (35/179; NM)	NM (50.3)	Delayed (4-6 mo)	Mx, Md	39 smokers	Some diabetic patients, but exact number not informed
Seki	2020	RA (unicenter)	Japan / University	35 (14/21; 13)	NM (66)	NM	Mx, Md	No	-
Simons	2015	RA (unicenter)	Belgium / University	185 (69/116; 50)	20-88 (56.4)	Delayed (3-6 mo)	Md	29 smokers	3 diabetic, 6 osteoporosis
Singh	2020	RA (unicenter)	India / University	826 (516/310; 96)	NM	NM	Mx, Md	124 smokers	108 diabetic
Wang	2020	PS (unicenter)	USA / University	49 (15/34; 5)	25-70 (46.8)	Immediate, Delayed (3 mo)	Mx, Md	1 light smoker	2 diabetic
Wu	2016	RA (unicenter)	Canada / Private practice	728 (375/353; 142)	18-93 (57.7)	Immediate, Delayed	Mx, Md	83 smokers	Some diabetic patients, but exact number not informed

NM – not mentioned; PS – prospective study; RA – retrospective study; mo – months

d. **Table S2. Quality assessment of the included studies, according to the National Institutes of Health (NIH).**

Study	Year	Was the study question or objective clearly stated?	Was the study population clearly and fully described, including a case definition?	Were the cases consecutive?	Were the subjects comparable?	Was the intervention clearly described?	Were the outcome measures clearly defined, valid, reliable, and implemented consistently across all study participants?	Was the length of follow-up adequate ? [a]	Were the statistical methods well-described?	Were the results well-described?	Total (n/9)
Alsaadi	2008a	1	1	1	1	1	1	1	1	1	9/9
Alsaadi	2008b	1	1	0	1	1	1	1	1	1	8/9
Altay	2018	1	1	0	1	1	1	1	0	1	7/9
Bertl	2019	1	1	1	1	0	1	1	1	0	7/9
Cabrera-Domínguez	2017	1	1	0	1	1	1	1	0	1	7/9
Chrcanovic	2017	1	1	1	1	1	1	1	1	1	9/9
Coskunses	2021	1	1	1	1	1	1	1	1	1	9/9
Grandi	2012a	1	1	1	1	1	1	1	1	1	9/9
Grandi	2012b	1	1	1	1	1	1	1	1	1	9/9
Krennmair	2016	1	1	0	1	1	1	1	1	1	8/9
Lee	2018	1	1	0	1	1	1	1	1	0	7/9
Lee	2019	1	1	0	1	1	1	1	1	1	8/9
Lobato	2020	1	1	0	1	1	1	1	1	0	7/9
Maló	2019a	1	1	1	1	1	1	1	1	1	9/9
Maló	2019b	1	1	0	1	1	1	1	1	1	8/9
Moy	2005	1	1	1	1	1	1	1	1	0	8/9
Park	2017	1	1	1	1	1	1	1	1	0	8/9
Park	2020	1	1	0	1	1	1	1	1	1	8/9
Schwartz-Arad	2016	1	1	1	1	1	1	1	1	1	9/9
Seki	2020	1	1	1	1	1	1	1	0	1	8/9
Simons	2015	1	1	0	1	1	1	1	1	1	8/9
Singh	2020	1	1	1	1	1	1	1	0	0	7/9
Wang	2020	1	1	0	1	1	1	1	1	1	8/9
Wu	2016	1	1	0	1	1	1	1	1	1	8/9

[a] 6 months of follow-up was chosen to be of adequate length.
